# Fungal Community Investigation from Propolis Natural Products: Diversity and Antibacterial Activities Evaluation

**DOI:** 10.1155/2022/7151655

**Published:** 2022-04-16

**Authors:** Souhir Sallemi, Abdelmalek Lekired, Nedra Korbi, Ilhem Saadouli, Ameur Cherif, Ines Zidi, Naouel Klibi, Hadda-Imene Ouzari, Amor Mosbah

**Affiliations:** ^1^LR03ES03 Laboratoire de Microorganismes et Biomolécules Actives, Faculté des Sciences de Tunis, Université Tunis Manar, Tunis 2092, Tunisia; ^2^Higher Institute for Biotechnology (ISBST), LR Biotechnology and Bio-Geo Resources Valorization, University of Manouba, BVBGR-LR11ES31, Biotechpole Sidi Thabet, Manouba 2020, Ariana, Tunisia

## Abstract

Discovering new species and interesting bioactive metabolites from customary sources is becoming progressively laborious. Propolis constitutes the largest diversified reserve of microbial constituents in the beehive. However, fungal communities associated with these environments remain insufficiently established. We present the first detailed investigation of the cultivable fungal community associated with Tunisian propolis, and we evaluate its antibacterial properties against pathogenic bacteria. A total of 80 fungal strains were isolated from propolis samples derived from seven different Tunisian locations. The majority of the isolated fungi were classified as *Ascomycota* (97.5%), and only 2.5% belonged to *Basidiomycota*. Our collection was clustered into 15 genera, among which *Coniochaeta* (36.25%), *Aspergillus* (15%), *Penicillium* (13.75%), *Cladosporium* (10%), *Fusarium* (7.5%), *Didymella* (5%), and *Alternaria* (3.75%) were the most common. Evaluation of the antibacterial activity revealed that 25.6% of the total community showed a broad range of antibacterial activity. Particularly, the *Penicillium griseofulvum* CC8 strain has manifested the strongest inhibitory effects against all the tested bacteria.

## 1. Introduction

Honey bees (*Apis mellifera*) play a vital role in preserving the health of natural and agricultural ecosystems [1]. They have complex interconnections with their environment and a diverse range of microorganisms [2]. Indeed, the honeybees and the hive environments are accommodated by various arrays of microbes [3]. The global hive microbial communities are captured from the foraging environment and the hive microenvironment [4]. They play a crucial role in honeybee protection during growth, development, and reproduction [5]. Grubbs et al. [6] suggest that microbial communities in hives are partitioned by their different components, including bees, propolis, honey, bee pollen, royal jelly, bee bread, and beeswax. To understand the hive-microbiota interaction, some research has focused on honey bee microbial gut inhabitants [7]. However, other hive components, particularly propolis, remain insufficiently established [8].

Propolis is a natural substance accumulated by honeybees from various plant sources and mixed with beeswax and salivary enzymes [9]. Honeybees use this resinous mixture as a defence mechanism against predators, as a protective effect against different pathogens, as a thermal and waterproof isolator, and as a means to mend corruption and to close open spaces in the beehive [10, 11]. It is considered among the most diversified natural substances due to the high complexity of its chemical composition. More than 300 constituents have been described in different propolis samples [12, 13]. The chemical compositions of propolis are dependent on the flora of each region, the environmental climate of the collection site, and the bees' genetic background [14, 15]. The nature of propolis compounds forms the basis for their great therapeutic properties. As a natural resinous material, propolis has been used as a traditional antidote for numerous sicknesses due to its biological and pharmacological properties [16]. It is historically considered as an excellent source of antimicrobial metabolites against a range of pathogens [17–21]. Propolis also displays antiviral activity against a large number of viruses. In particular, it can represent a low-cost cure as a potential inhibitor of SARS-CoV-2 in the oropharyngeal niche [22, 23]. Similarly, it could conceivably be used as an antiinflammatory [24, 25], antioxidant [26], and anticancer agent [27], in addition to a reported antiprotozoal effect [28, 29]. In recent years, the roles of propolis-associated microbes are increasingly studied [30–32]. Also, it has been revealed that propolis has the highest microbial community richness among hive components [6].

However, many factors contribute to the high diversity in the composition and structure of the propolis microbiome. The food availability during the foraging season could change the microbial communities living in the beehives [33, 34]. In addition, the global hive microbial communities can also be influenced by some compounds used in agricultural treatments such as thiacloprid, nitenpyram, chlorothalon, imidacloprid, and coumaphos [2, 35–37]. These communities are further influenced by the geographical origin, including the vegetation type from the respective areas [38].

In spite of the fact that propolis constitutes the largest diversified reserve of the microbial constituents in the beehive, fungal communities associated with these environments remain insufficiently established. In this context, the purpose of the current study was to isolate and identify fungal communities from Tunisian propolis samples and to evaluate their antibacterial properties against pathogenic bacteria.

## 2. Materials and Methods

### 2.1. Propolis Sampling and Fungal Isolation

**Figure 1:**
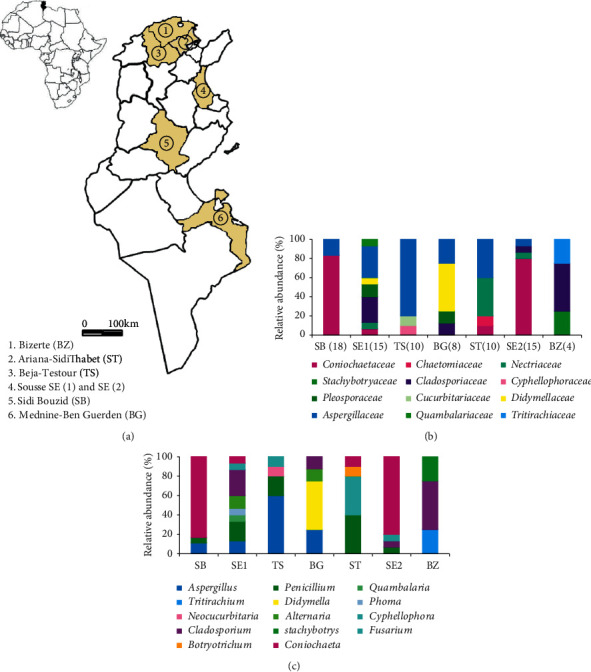
(a)Geographic localization of Tunisian propolis samples, (b) family-level distributions and the total number of isolates in the site is given between parentheses, and (c) genus-level distribution of the fungal community among the studied sites.

Propolis samples were collected from different geographic locations in Tunisia: Ben Guerdane (BG), Sidi Bouzid (SB), Bizerte (BZ), Testour (TS), Sidi Thabet (ST), Sousse 1 (SE1), and Sousse 2 (SE2) (Figure 1(a)). Fungal isolation was performed by the suspension-dilution method. In brief, decimal dilutions (10^−1^ to 10^−6^) were prepared from the initial sample solution. Then, 0.1 ml of each suspension was rolled out on the surface of a potato dextrose agar (PDA) medium. The plates were incubated at 28°C for 7 days. Fungal isolates were purified following successive subcultures of the colonies using the same medium.

### 2.2. DNA Extraction, PCR Amplification, and Sequencing

DNA was extracted following Liu et al. [39].In brief, fresh mycelia were mixed with 500 *μ*L of lysis buffer (Triton X-100 2% v/*v*, SDS 1% p/v, NaCl 100 mM, Tris-HCl 10 mM pH = 8, EDTA 1 mM) and incubated for 30 min. Potassium acetate buffer (150 *μ*L) was added to the mixture, followed by centrifugation at 12000*g*. The supernatant was mixed with twice the volume of isopropanol followed by centrifugation; finally, the pellet was washed with a 70% ethanol solution. The microtubes were centrifuged at 12000*g* for 5 min. The pellet was resuspended in 40–50 *μ*L of TE buffer (Tris-HCL 25 mM, pH = 8,0; EDTA 0,5 mM) and stored at −20°C until used. DNA was quantified by measuring the absorbance at 260 nm (A260) and 280 nm (A280) using the NanoDrop spectrophotometer 2000 (ThermoFisher Scientific). Then, the nuclear ribosomal internal transcribed spacer (ITS) region was amplified using the primer pair ITS1/ITS4 [40]. For all PCR runs, electrophoresis was performed in 1.5% agarose gel with ethidium bromide and visualized under UV light.

PCR products were purified using the QIAquick Wizard PCR purification Kit (Promega) according to the manufacturer's instructions. The sequences were determined by cycle sequencing using the Taq Dye Deoxy Terminator Cycle Sequencing kit (Applied Biosystems) and fragment separation in an ABI Prism™ 3130 DNA sequencer (Applied Biosystems).

### 2.3. Fungal Identification and Functional Profile Analysis

The sequence quality was checked in DECIPHER V. 2.20.0. The high-quality ITS sequences (5.8S region) were compared with the GenBank database [41] using the basic local alignment search tool (BLAST) algorithm [42]. Furthermore, the BLAST hit list result was thoroughly examined. In order to retrieve the accession numbers of the species hypotheses (SHs) of UNITE [43], the sequences were compared with the ITS-based species hypotheses in UNITE. The clustered sequences based on their SHs were then used to obtain the functional profile of the recovered community using the FUNGuild database [44].

### 2.4. Fermentation and Antibacterial Activity Determination

Fermentations were carried out in 250 mL Erlenmeyer flasks containing 50 mL of 2% malt extract medium at 28°C and 120 rpm for five to seven days. After fermentation, the supernatants were filtered, concentrated, and used to assess their antibacterial activities. The antibacterial activities of the isolates were evaluated by Radial Diffusion Assay (RDA) according to the method previously described by Lehrer [45], using the following: three Gram-positive bacterial strains, viz. *Staphylococcus aureus*, *Enterococcus faecalis* (MK584170), and *Bacillus cereus* (NR074540.1) and the two Gram-negative bacterial strains *Escherichia fergusonii* (MK584171) and *Salmonella enterica* (MK584173). All strains were selected for their wide pathological impact and their significant resistance to antimicrobial agents of public health. Indicator strains were obtained from the microbial collections of the Laboratory of Microorganisms and Active Biomolecules (LMBA), Faculty of Sciences of Tunis. All the statistical analyses were performed using RStudio (4.0).

## 3. Results

### 3.1. Identification of Cultivable Fungi Derived from Propolis Samples

A total of 80 strains were isolated from seven propolis samples collected from six different geographical locations (Figure 1(a)). The majority of isolated fungi were classified as *Ascomycota* (97.5%) of the three classes of *Sordariomycetes* (46.25%), *Dothideomycetes* (21.25%), and *Eurotiomycetes* (30%). In addition, two classes (*Exobasidiomycetes* (1.25%) and *Tritirachiomycetes* (1.25%)) belonged to the phylum *Basidiomycota* (2.5%).

The fungi we recovered were found to represent a total of 12 families (Figure 1(b)). It was determined that *Coniochaetaceae*, *Aspergillaceae*, *Cladosporiaceae*, *Nectriaceae*, *Didymellaceae*, and *Pleosporaceae* were the most dominant families with 36.25%, 28.75%, 10%, 7.5%, 6.25%, and 3.75%, respectively. A total of 7.5% of the individuals exhibited a lower abundance with 1.25% for each family, namely, *Chaetomiaceae*, *Stachybotryaceae*, *Cyphellophoraceae*, *Cucurbitariaceae*, *Quambalariaceae*, and *Tritirachiaceae.*

In addition, multiple sequence alignments revealed a total of 15 different genera (Figure 1(c)). The most common genera were as follows: *Coniochaeta* (36.25%), *Aspergillus* (15%), *Penicillium* (13.75%), *Cladosporium* (10%), *Fusarium* (7.5%), *Didymella* (5%), and *Alternaria* (3.75%), which represent 91.25% of the total cultivable fungal diversity in the samples. Seven genera accounted for 8.75% of the total community, with 1.25% for each genus, namely, *Botryotrichum*, *Stachybotrys*, *Cyphellophora*, *Neocucurbitaria*, *Phoma*, *Quambalaria*, and *Tritirachium*.

### 3.2. Comparison of Fungal Community Composition from Different Geographic Locations

The highest number of different fungi was isolated from the SB site with 18 strains (22.5%; 18/80), but this showed the lowest diversity with three different genera corresponding to only two families, of which *Coniochaeta* (83.3%; 15/18) was the most abundant genus. The lowest number of fungi was isolated from the BZ site. These strains corresponded to *Cladosporium*, *Stachybotrys*, and *Tritirachium* with abundance rates of (50%; 2/4), (25%; 1/4), and (25%; 1/4), respectively. Among the seven studied sites, SE1 represented the most diversified site. These fungi represented eight different genera from a total of seven families. In relation to the frequency and distribution of fungal taxa according to the sites, the genus *Cladosporium* was the most commonly isolated (26.7%; 4/15).

In relation to the frequency and distribution of fungal taxa across the sites, some genera were specific to only one or two sites. For instance, *Botryotrichum* (ST), *Cyphellophora* (TS), *Neocucurbitaria* (TS), *Stachybotrys* (BZ), *Phoma* (SE1), *Quambalaria* (SE1), and *Tritirachium* (BZ) were isolated from only one site. On the other hand, *Alternaria* genus was found in both SE1 and BG locations. Similarly, *Coniochaeta*, *Fusarium*, *Cladosporium*, *Aspergillus*, and *Penicillium* were found in the majority of studied sites (4-5 sites).

### 3.3. Functional Profile Analysis

**Figure 2:**
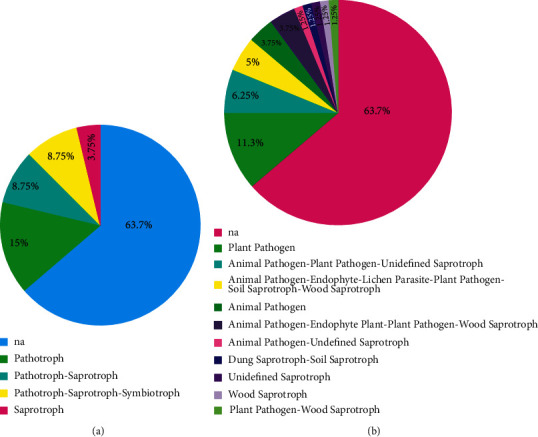
FUNGuild database results of the cultivable fungal community associated with propolis, (a) distribution of the fungal community by trophic mode, and (b) distribution of the fungal community based on the functional category of guild.

The corresponding species hypotheses and their digital object identifiers (DOIs) are specified in the supplementary table. The clustered sequences based on their SHs produced 20 operational taxonomic units (OTUs; [46]). The FUNGuild results showed a prevalence of pathotrophs, saprotrophs, and/or symbiotrophs as the trophic mode and plant-animal-pathogen and wood-soil-saprotroph on the functional category of guild (among 12 identified OTUs at trophic mode and guild levels) (Figures 2(a) and 2(b)).

### 3.4. Antibacterial Activity of Fungal Strains

**Figure 3:**
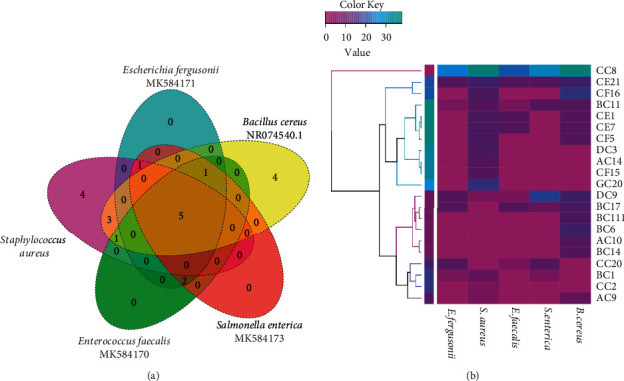
Antibacterial activity of isolated fungi, (a) Venn diagram showing the number of active isolates against unique and shared pathogenic bacteria, and (b) heatmap summarising variation in the inhibition diameter (mm) of active fungi.

As reported in Figures 3(a) and 3(b), 21 isolates (accounting for 26.25%) were able to inhibit one or more of the indicator bacteria. The other 59 strains (73.75%) did not show inhibitory activities. The isolated strains exhibited higher antibacterial activity against Gram-positive bacteria (*B. cereus*, *S. aureus*, and *E. faecalis*) than against Gram-negative bacteria (*E. fergusonii* and *S. enterica*), with 21 and 7 strains, respectively. Among them, four isolates exhibited broad-spectrum antibacterial activity towards all the tested bacteria, including *Penicillium griseofulvum* (CC8), *Alternaria* sp (DC9), *Penicillium griseoroseum* (BC11), and *Penicillium camemberti* (CE21). Among the active isolates, *P. griseofulvum* CC8 showed the strongest inhibitory effects against all the tested bacteria with inhibition zone diameters ranging from 24 to 38 mm. Fifteen isolates (71.42%; 15/21) among the active strains showed antibacterial activity against *S. aureus* with an inhibition zone diameter ranging from 3 to 38 mm. Activity against *E. fergusonii* and *S. enterica* was found in seven strains among active strains (33.3%; 7/21), especially the strain CC8 of *P. griseofulvum*, which has an inhibition zone of 26 and 30 mm, respectively. A total of eight strains (38.1% of all active isolates, 8/21) inhibited *E. faecalis* with an inhibition zone diameter ranging from 3 to 24 mm. Fourteen strains (66.7%; 14/21) displayed inhibitory activity against *B. cereus*.

## 4. Discussion

Among the various components of the beehive, the propolis (and particularly the associated fungal community) is still poorly studied, despite being considered as the largest diversified reserve of the microbial community in the hive [6]. Discovering new species and interesting bioactive compounds from customary sources is becoming progressively laborious. Among the few studies being conducted to study propolis microbial communities, only the work of De Souza et al. [30] provided insights into fungi living in propolis. Here, we describe the first detailed investigation of the cultivable fungal community associated with Tunisian propolis samples based on a cultivation approach.

The obtained fungal community was composed of 12 families, 14 genera, 9 orders, five classes, and two phyla, revealing that propolis harbors a varied fungal community. The same trend was reported by other researchers, confirming that propolis, often thought to be relatively aseptic, hosted a very large number of fungi [6]. It is also clearly demonstrated that propolis is a complex ecosystem providing a substantial fungal diversity. Our findings showed that *Ascomycota* was the most abundant phylum (97.6%). The majority of the isolates found in our propolis samples belonged to the genera *Coniochaeta, Aspergillus*, *Penicillium*, *Cladosporium*, and *Fusarium*. Among the dominant genera in the obtained collection*, Coniochaeta* is characterized by the ability to grow in an acidic environment [47] and members of this genus have been previously isolated from young plants [48], floral nectar [49], and fruit trees [50]. In this way, their origin could be attributed to honeybees' gut microbiota as a source of acidophilic microorganisms and/or from plant sources during the foraging season. Furthermore, the genera *Aspergillus*, *Penicillium*, and *Fusarium* are widespread in various environments, including soil, air, and vegetation. Their origin may be also linked to plant materials and/or compounds that are fortuitously imported during the formulation and collection of propolis.

In accordance with the study of De Souza et al. [30], the genus *Penicillium* was also found abundantly in propolis, particularly, *P. citrinum*, *P. crustosum*, *P. fasciculado*, *P. janthinellum*, and *P. purpurogenum*. The genera *Aspergillus* (0.1–43%), *Cladosporium* (0.65–4.9%), and *Fusarium* (0.14%) have already been reported in propolis samples, using ultra-high-throughput marker gene sequencing [51]. The genus *Cladosporium* was also detected as the most abundant genus from the phylum *Ascomycota* (52.20%) in corbicular pollen and hive-stored bee bread, in addition to the genus *Penicillium* (2.55%), *Aspergillus* (2.00%), and *Alternaria* (1.93%) [52].

The propolis samples from the seven different regions appeared to be very different from each other. Thus, the composition of the propolis microbiota may follow a geographical trend. This diversity could conceivably be attributed to various factors associated with the investigated regions, given that the geographical origin of propolis is defined by plant sources from respective areas [38]. Consequently, fungus-associated propolis variability might be explained by the nature of the local flora at the site of collection [53]. In addition, food accessibility during the foraging season could change the microbial communities living in the beehives [34]. Moreover, the variability of the fungal community could be influenced by agricultural treatments [37]. Due to their fermentation features, honeybees' gut microbiota can affect the conversion of plant buds and exudates into propolis, particularly the fungal propolis community [2, 54].

The FUNGuild results showed that the fungal community was composed of 10 functional guild categories and 4 different trophic modes. As compared to GenBank results, the strains belonging to *Cladosporium*, *Coniochaeta*, and *Lecythophora* genera did not give the same identification in UNITE analysis (Supplementary Table). This can be due to the limitations of using GenBank BLAST search, as around 27% of GenBank fungal ITS sequences were submitted with incorrect taxonomic identification [55]. Furthermore, fungal identification using the ITS marker is unsatisfactory since it lacks enough variation to differentiate between species in some groups of fungi [56]. More research based on the combination of multiple molecular markers is required to confirm species' fungal identification. As a result, various markers are used to identify species. The highest resolution for identifying *Coniochaeta* species is provided by TEF-1 (translation elongation factor 1-alpha) [57–59]. Furthermore, the beta-tubulin (tub2/BenA) and second largest subunits of RNA polymerase (RPB2) genes are highly favored in the identification of *Aspergillus* species [60]. For accurately identifying *Penicillium* species, the BenA can successfully be used [61]. The TEF-1*α*, the RPB1/RPB2, and the partial actin were used for species-level identification of *Fusarium* and *Cladosporium* [62, 63].

The FUNGuild results (Figures 2(a) and 2(b)) for the cultivable fungal community associated with propolis showed that our fungal community reflected plant-animal-pathogen and wood-soil-saprotroph fungal life styles [44], confirming that the propolis microbiota origin could be attributed to the environmental sampling and the microbial communities existing in the beehives (honeybees' gut).

Concerning the production of active compounds, 25.6% of the total community members indicated a broad range of antibacterial activity (Figure 3(a)), revealing the production of various bioactive substances. In accordance with previous studies, it was noticed that the detected antibacterial activity was higher against Gram-positive bacteria than against Gram-negative bacteria [24, 64]. Most of the active strains belonging to the genera *Aspergillus*, *Penicillium*, and *Fusarium* are historically known for their significant antibacterial potential and were recently described to generate new antibacterial compounds (Table 1). In fact, these genera are fast-growing species, are easily obtained from many substrates, and are able to grow in extreme environments [65–67].

Among these strains, *P. griseofulvum* CC8 revealed the strongest inhibitory effects against all the tested bacteria (Figure 3(b)). As previously described, *P. griseofulvum* has been shown to be a rich source of interesting bioactive products with diverse features including griseofulvin derivatives and indole alkaloids exhibiting anti-HIV activities [68], Penifulvin A with antiinsect activity [69], polyketide metabolites displaying antitumoral activity against prostatic carcinoma cells (PC-3), and penigrisacid *D*, which proved cytotoxicity against ECA-109 tumor cells and particularly antimicrobial activities against *Staphylococcus aureus* and *Bacillus subtilis* [70, 71]. The genus *Coniochaeta*, too, is well known for its antimicrobial capabilities. For instance, *Coniochaeta ellipsoidea* (DSM 13856) produces coniosetin, a novel antibiotic with noticeable antibacterial and antifungal properties [72], and *C. saccardoi* was shown to form two new antifungal compounds: coniochaetones A and B [73]. Furthermore, *Coniochaeta* sp. was found to have significant activity against *Enterococcus faecium* and *Enterococcus faecalis* through coniothiepinols A production [74]. Thus, propolis' antimicrobial activity could be due to the antibacterial compounds produced by the associated propolis microorganisms, especially the fungal communities, which may contribute to the intrinsic protective role exerted by propolis against parasites and pathogens. Further analyses are needed to elucidate the different biological compounds nature of the selected strains.

## 5. Conclusions

In the present study, we have shown that bee propolis hosts a significant number of cultivable fungi, with *Ascomycota* as the most abundant phylum. (i) Samples from different geographical locations appeared to be very different from each other, confirming that the microbial community of this resinous mixture followed a geographical trend. (ii) The FUNGuild results showed that the propolis microbiota origin could be attributed to the environmental sampling and the microbial communities existing in the beehives. (iii) 25.6% of the total community showed a broad range of antibacterial activity. In particular, the strain *Penicillium griseofulvum* CC8 was found to have the strongest inhibitory effects against all the tested bacteria.

## Figures and Tables

**Table 1:** Recent novel published antibacterial molecules from the genera *Penicillium*, *Aspergillus,* and *Fusarium*.

Strain	Novel published antibacterial molecule	Pathogenic tested bacteria	Reference
*Penicillium spathulatum* Em19	Spathullin A and B	*E. coli*, *Acinetobacter baumannii*, *Enterobacter cloacae*, *Klebsiella pneumonia*, *P. aeruginosa*, and *S. aureus*	[75]
*Penicillium* sp. HDN151272	Ketidocillinones B and C	*P. aeurigenosa*, *Mycobacterium phleiand* MRCNS (methicillin-resistant coagulase-negative *staphylococci*)	[76]
*Aspergillus* sp. DM94	Pyrones (1 and 6)	*Helicobacter pylori*	[77]
*Aspergillus* sp. YHZ-1	Asperphenone A and B	*S. aureus* CMCC(B) 26003, *Streptococcus pyogenes* (ATCC19615), *B. subtilis* CICC 10283, and *Micrococcus luteus*	[78]
*Fusarium napiforme*	6-Hydroxy-astropaquinone B (1) and astropaquinone *D* (2)	*S. aureus* (NBRC 13276) and *P. aeruginosa* (ATCC 15442)	[79]
*Fusarium* sp.	3-Epi-fusarielin H, 3-O-methyl-fusarielin H and 3-O-methylepi-fusarielin H	*S. aureus* (NBRC 13276)	[80]
*Fusarium solani* JK10	7–Desmethyl fusarin C derivatives (1–7)	*E. coli* (DSM 1116)	[81]

## Data Availability

The nucleotide sequences data used to support the findings of this study are publicly available in the GenBank repository at the National Center for Biotechnology Information (NCBI) (https://www.ncbi.nlm.nih.gov/genbank/). All data are included in Results and Discussion in this paper. Fungal ITS sequences were deposited in the GenBank database, and their accession numbers are listed in the supplementary table.

## Supplementary Materials

Supplementary Materials Table: UNITE and GenBank analysis.
