# Supplementary material for: Fungal Community Investigation from Propolis Natural Products: Diversity and Antibacterial Activities Evaluation
Source: Evid Based Complement Alternat Med. 2022 Apr 16;2022:7151655. doi: 10.1155/2022/7151655 (PMC9034937; doi:10.1155/2022/7151655)
Supplement: Supplementary Materials — Table: UNITE and GenBank analysis. [file 7151655.f1.docx]

**Table:** UNITE and GenBank analysis

| Identifier | Accession Numbers | UNITE | | | GenBank | | |
| --- | --- | --- | --- | --- | --- | --- | --- |
|  |  | **SH** | **DOI** | **Unite Taxonomy** | **GenBank Taxonomy** | **Coverage**  **(%)** | **Similarity (%)** |
| CC20 | MZ451225 | SH1649133.08FU | dx.doi.org/10.15156/BIO/SH1649133.08FU | *Aspergillus jensenii* | *Aspergillus jensenii* | 100 | 100 |
| AC8 | MZ451222 | SH1549902.08FU | dx.doi.org/10.15156/BIO/SH1549902.08FU | *Aspergillus insuetus* | *Aspergillus insuetus* | 100 | 99,79 |
| DC8 | MZ451223 | SH1549903.08FU | dx.doi.org/10.15156/BIO/SH1549903.08FU | *Aspergillus unguis* | *Aspergillus unguis* | 100 | 100 |
| CC2 | MZ451224 | SH1649133.08FU | dx.doi.org/10.15156/BIO/SH1649133.08FU | *Aspergillus creber* | *Aspergillus creber* | 100 | 100 |
| DC10 | MZ451226 | SH1549900.08FU | dx.doi.org/10.15156/BIO/SH1549900.08FU | *Aspergillus versicolor* | *Aspergillus versicolor* | 100 | 100 |
| AC9 | MZ451227 | SH1549902.08FU | dx.doi.org/10.15156/BIO/SH1549902.08FU | *Aspergillus insuetus* | *Aspergillus insuetus* | 100 | 100 |
| BC1 | MZ451228 | SH1549902.08FU | dx.doi.org/10.15156/BIO/SH1549902.08FU | *Aspergillus insuetus* | *Aspergillus insuetus* | 99 | 100 |
| CC6 | MZ451230 | SH1649133.08FU | dx.doi.org/10.15156/BIO/SH1649133.08FU | *Aspergillus creber* | *Aspergillus creber* | 100 | 100 |
| CC9 | MZ451231 | SH1549900.08FU | dx.doi.org/10.15156/BIO/SH1549900.08FU | *Aspergillus versicolor* | *Aspergillus versicolor* | 98 | 100 |
| CC12 | MZ451232 | SH1549900.08FU | dx.doi.org/10.15156/BIO/SH1549900.08FU | *Aspergillus unguis* | *Aspergillus* sp | 100 | 100 |
| BC14 | MZ451233 | SH1549902.08FU | dx.doi.org/10.15156/BIO/SH1549902.08FU | *Aspergillus insuetus* | *Aspergillus insuetus* | 100 | 100 |
| CC3 | MZ451229 | SH1549900.08FU | dx.doi.org/10.15156/BIO/SH1549900.08FU | *Aspergillus* | *Aspergillus* sp | 100 | 100 |
| BB19 | MZ449225 | SH1572792.08FU | dx.doi.org/10.15156/BIO/SH1572792.08FU | *Cladosporium halotolerans* | *Cladosporium halotolerans* | 99 | 100 |
| BC7 | MZ449226 | SH1572792.08FU | dx.doi.org/10.15156/BIO/SH1572792.08FU | *Cladosporium halotolerans* | *Cladosporium halotolerans* | 100 | 99,79 |
| CF3 | MZ449227 | SH1572792.08FU | dx.doi.org/10.15156/BIO/SH1572792.08FU | Envir Eukaryota | *Cladosporium* sp | 98 | 100 |
| GC27 | MZ449228 | SH1572792.08FU | dx.doi.org/10.15156/BIO/SH1572792.08FU | *Cladosporium cladosporioides* | *Cladosporium cladosporioides* | 100 | 100 |
| BC12 | MZ449229 | SH1572792.08FU | dx.doi.org/10.15156/BIO/SH1572792.08FU | *Cladosporium halotolerans* | *Cladosporium halotolerans* | 100 | 100 |
| DC12 | MZ449230 | SH1572792.08FU | dx.doi.org/10.15156/BIO/SH1572792.08FU | *Cladosporium halotolerans* | *Cladosporium* sp | 100 | 100 |
| BC15 | MZ449231 | SH1572792.08FU | dx.doi.org/10.15156/BIO/SH1572792.08FU | *Cladosporium halotolerans* | *Cladosporium halotolerans* | 99 | 100 |
| GC20 | MZ449232 | SH1572792.08FU | dx.doi.org/10.15156/BIO/SH1572792.08FU | *Cladosporium sphaerospermum* | *Cladosporium sphaerospermum* | 100 | 100 |
| DC6 | MZ449313 | SH1547057.08FU | dx.doi.org/10.15156/BIO/SH1547057.08FU | *Didymella microchlamydospora* | *Didymella microchlamydospora* | 100 | 100 |
| DC1 | MZ449314 | SH1547057.08FU | dx.doi.org/10.15156/BIO/SH1547057.08FU | *Didymella microchlamydospora* | *Didymella microchlamydospora* | 100 | 100 |
| DC3 | MZ449315 | SH1547057.08FU | dx.doi.org/10.15156/BIO/SH1547057.08FU | *Didymella microchlamydospora* | *Didymella microchlamydospora* | 99 | 100 |
| DC2 | MZ449316 | SH1547057.08FU | dx.doi.org/10.15156/BIO/SH1547057.08FU | *Didymella calidophila* | *Didymella calidophila* | 100 | 100 |
| BC9 | MZ448580 | SH1546388.08FU | dx.doi.org/10.15156/BIO/SH1546388.08FU | *Fusarium* | *Fusarium* sp | 100 | 100 |
| CE7 | MZ448581 | SH1610345.08FU | dx.doi.org/10.15156/BIO/SH1610345.08FU | *Fusarium napiforme* | *Fusarium oxysporum* | 99 | 100 |
| CF18 | MZ448582 | SH1656686.08FU | dx.doi.org/10.15156/BIO/SH1656686.08FU | *Envir Fusarium* | *Fusarium oxysporum* | 100 | 100 |
| CE1 | MZ448583 | SH1656686.08FU | dx.doi.org/10.15156/BIO/SH1656686.08FU | *Fusarium oxysporum* | *Fusarium oxysporum* | 95 | 100 |
| CE13 | MZ448584 | SH1610345.08FU | dx.doi.org/10.15156/BIO/SH1610345.08FU | *Fusarium* | *Fusarium* sp | 99 | 100 |
| CE15 | MZ448585 | SH1656686.08FU | dx.doi.org/10.15156/BIO/SH1656686.08FU | *Fusarium oxysporum* | *Fusarium oxysporum* | 100 | 100 |
| AC5_1 | MZ448479 | SH1645097.08FU | dx.doi.org/10.15156/BIO/SH1645097.08FU | Envir Eukaryota | *Coniochaeta* sp | 99 | 99,5 |
| AC14 | MZ448480 | SH1645097.08FU | dx.doi.org/10.15156/BIO/SH1645097.08FU | Envir Eukaryota | *Lecythophora* sp | 100 | 99,57 |
| CF2 | MZ448481 | SH1645097.08FU | dx.doi.org/10.15156/BIO/SH1645097.08FU | Envir Eukaryota | *Coniochaeta* sp | 100 | 100 |
| CF7 | MZ448482 | SH1645097.08FU | dx.doi.org/10.15156/BIO/SH1645097.08FU | Envir Eukaryota | *Coniochaeta* sp | 99 | 100 |
| CF15 | MZ448483 | SH1645097.08FU | dx.doi.org/10.15156/BIO/SH1645097.08FU | Envir Eukaryota | *Lecythophora* sp | 97 | 100 |
| AC2_1 | MZ448484 | SH1645097.08FU | dx.doi.org/10.15156/BIO/SH1645097.08FU | Envir Eukaryota | *Coniochaeta* sp | 99 | 100 |
| AC2 | MZ448485 | SH1645097.08FU | dx.doi.org/10.15156/BIO/SH1645097.08FU | Envir Eukaryota | *Coniochaeta* sp | 100 | 100 |
| AC3 | MZ448486 | SH1645097.08FU | dx.doi.org/10.15156/BIO/SH1645097.08FU | Envir Eukaryota | *Coniochaeta* sp | 99 | 100 |
| AC12 | MZ448487 | SH1645097.08FU | dx.doi.org/10.15156/BIO/SH1645097.08FU | Envir Eukaryota | *Lecythophora* sp | 81 | 100 |
| AC13_2 | MZ448488 | SH1645097.08FU | dx.doi.org/10.15156/BIO/SH1645097.08FU | Envir Eukaryota | Coniochaeta sp | 100 | 100 |
| AC13 | MZ448489 | SH1645097.08FU | dx.doi.org/10.15156/BIO/SH1645097.08FU | Envir Eukaryota | *Coniochaeta* sp | 100 | 100 |
| AC14_2 | MZ448490 | SH1645097.08FU | dx.doi.org/10.15156/BIO/SH1645097.08FU | Envir Eukaryota | *Coniochaeta* sp | 99 | 100 |
| BC13_1 | MZ448491 | SH1645097.08FU | dx.doi.org/10.15156/BIO/SH1645097.08FU | Envir Eukaryota | *Coniochaeta* sp | 99 | 100 |
| CF12 | MZ448492 | SH1645097.08FU | dx.doi.org/10.15156/BIO/SH1645097.08FU | Envir Eukaryota | *Lecythophora* sp | 100 | 100 |
| CF16_1 | MZ448493 | SH1645097.08FU | dx.doi.org/10.15156/BIO/SH1645097.08FU | Envir Eukaryota | *Coniochaeta* sp | 100 | 99,77 |
| AC15_1 | MZ448494 | SH1645097.08FU | dx.doi.org/10.15156/BIO/SH1645097.08FU | Envir Eukaryota | *Coniochaeta* sp | 99 | 100 |
| CF13 | MZ448495 | SH1645097.08FU | dx.doi.org/10.15156/BIO/SH1645097.08FU | Envir Eukaryota | *Coniochaeta* sp | 99 | 100 |
| AC1 | MZ448496 | SH1645097.08FU | dx.doi.org/10.15156/BIO/SH1645097.08FU | Envir Eukaryota | *Coniochaeta* sp | 99 | 100 |
| AC4 | MZ448497 | SH1645097.08FU | dx.doi.org/10.15156/BIO/SH1645097.08FU | Envir Eukaryota | *Coniochaeta* sp | 99 | 100 |
| AC5 | MZ448498 | SH1645097.08FU | dx.doi.org/10.15156/BIO/SH1645097.08FU | Envir Eukaryota | *Lecythophora* sp | 100 | 99,58 |
| AC16 | MZ448499 | SH1645097.08FU | dx.doi.org/10.15156/BIO/SH1645097.08FU | Envir Eukaryota | *Lecythophora* sp | 99 | 99,79 |
| CE16_1 | MZ448500 | SH1645097.08FU | dx.doi.org/10.15156/BIO/SH1645097.08FU | Envir Eukaryota | *Lecythophora* sp | 98 | 100 |
| CF5 | MZ448501 | SH1645097.08FU | dx.doi.org/10.15156/BIO/SH1645097.08FU | *Coniochaeta* | *Coniochaeta* sp | 99 | 99,58 |
| CF9 | MZ448502 | SH1645097.08FU | dx.doi.org/10.15156/BIO/SH1645097.08FU | Envir Eukaryota | *Lecythophora* sp | 98 | 100 |
| CF10 | MZ448503 | SH1645097.08FU | dx.doi.org/10.15156/BIO/SH1645097.08FU | Envir Eukaryota | *Coniochaeta* sp | 97 | 99,79 |
| CF11 | MZ448504 | SH1645097.08FU | dx.doi.org/10.15156/BIO/SH1645097.08FU | Envir Eukaryota | *Lecythophora* sp | 98 | 99,79 |
| CF16 | MZ448505 | SH1645097.08FU | dx.doi.org/10.15156/BIO/SH1645097.08FU | Envir Eukaryota | *Lecythophora* sp | 98 | 100 |
| AC13_1 | MZ448506 | SH1645097.08FU | dx.doi.org/10.15156/BIO/SH1645097.08FU | Envir Eukaryota | *Lecythophora* sp | 99 | 99,18 |
| CF6 | MZ448507 | SH1645097.08FU | dx.doi.org/10.15156/BIO/SH1645097.08FU | Envir Eukaryota | *Lecythophora* sp | 99 | 99,39 |
| AC10 | MZ449111 | SH1529984.08FU | dx.doi.org/10.15156/BIO/SH1529984.08FU | *Penicillium* | *Penicillium dipodomyicola* | 100 | 99,55 |
| BC8 | MZ449112 | SH1529984.08FU | dx.doi.org/10.15156/BIO/SH1529984.08FU | *Penicillium aurantiogriseum* | *Penicillium* sp | 100 | 99,78 |
| BC11 | MZ449113 | SH1529984.08FU | dx.doi.org/10.15156/BIO/SH1529984.08FU | *Envir Penicillium* | *Penicillium griseoroseum* | 100 | 100 |
| BC111 | MZ449114 | SH1529998.08FU | dx.doi.org/10.15156/BIO/SH1529998.08FU | *Penicillium sanguifluum* | *Penicillium sanguifluum* | 100 | 100 |
| CF1 | MZ449115 | SH1529984.08FU | dx.doi.org/10.15156/BIO/SH1529984.08FU | *Metarhizium* | *Metarhizium* sp | 100 | 100 |
| CE13_1 | MZ449116 | SH1529989.08FU | dx.doi.org/10.15156/BIO/SH1529989.08FU | *Penicillium corylophilum* | *Penicillium corylophilum* | 99 | 100 |
| CC5 | MZ449117 | SH1529989.08FU | dx.doi.org/10.15156/BIO/SH1529989.08FU | *Penicillium corylophilum* | *Penicillium corylophilum* | 100 | 100 |
| CC8 | MZ449118 | SH1529984.08FU | dx.doi.org/10.15156/BIO/SH1529984.08FU | *Penicillium dipodomyicola* | *Penicillium griseofulvum* | 99 | 100 |
| CE3 | MZ449119 | SH1529984.08FU | dx.doi.org/10.15156/BIO/SH1529984.08FU | *Penicillium* | *Penicillium chrysogenum* | 100 | 100 |
| CE12 | MZ449120 | SH1529989.08FU | dx.doi.org/10.15156/BIO/SH1529989.08FU | *Penicillium corylophilum* | *Penicillium corylophilum* | 100 | 100 |
| CE21 | MZ449121 | SH1529984.08FU | dx.doi.org/10.15156/BIO/SH1529984.08FU | *Penicillium chrysogenum* | *Penicillium camemberti* | 100 | 100 |
| BC13 | MZ450145 | SH1547057.08FU | dx.doi.org/10.15156/BIO/SH1547057.08FU | *Ampelomyces* | *Phoma sojicola* | 99 | 100 |
| BC17 | MZ450146 | SH1526398.08FU | dx.doi.org/10.15156/BIO/SH1526398.08FU | *Alternaria* | *Ulocladium* sp | 100 | 99,8 |
| CC1 | MZ450147 | SH1636089.08FU | dx.doi.org/10.15156/BIO/SH1636089.08FU | *Cyphellophora* | *Cyphellophora olivacea* | 100 | 100 |
| CE6 | MZ450148 | SH1615603.08FU | dx.doi.org/10.15156/BIO/SH1615603.08FU | Fungi | *Botryotrichum murorum* | 99 | 100 |
| DC9 | MZ450149 | SH1526398.08FU | dx.doi.org/10.15156/BIO/SH1526398.08FU | *Alternaria* | *Alternaria* sp | 100 | 100 |
| GC25 | MZ450150 | SH1574224.08FU | dx.doi.org/10.15156/BIO/SH1574224.08FU | *Tritirachium oryzae* | *Tritirachium oryzae* | 97 | 100 |
| GC26 | MZ450151 | SH1557965.08FU | dx.doi.org/10.15156/BIO/SH1557965.08FU | Fungi | *Stachybotrys chlorohalonata* | 100 | 99,8 |
| CC7 | MZ450152 | SH1614483.08FU | dx.doi.org/10.15156/BIO/SH1614483.08FU | *Neocucurbitaria* | *Neocucurbitaria keratinophila* | 99 | 99,77 |
| BC15P | MZ450153 | SH1552035.08FU | dx.doi.org/10.15156/BIO/SH1552035.08FU | *Quambalaria cyanescens* | *Quambalaria cyanescens* | 100 | 100 |
| BC6 | MZ450154 | SH1526398.08FU | dx.doi.org/10.15156/BIO/SH1526398.08FU | *Ulocladium consortiale* | *Alternaria sorghi* | 99 | 100 |
